# Analysis of Bioactive Aroma Compounds in Essential Oils from Algerian Plants: Implications for Potential Antioxidant Applications

**DOI:** 10.3390/foods13050749

**Published:** 2024-02-28

**Authors:** Anis Bertella, Georgiana-Luminita Gavril, Magdalena Wrona, Davinson Pezo, Abouamama Sidaoui, Kheira Benlahcen, Mebrouk Kihal, Ewa Olewnik-Kruszkowska, Jesús Salafranca, Cristina Nerín

**Affiliations:** 1 Department of Molecular and Cellular Biology, Faculty of Life and Nature Sciences, Abbes Laghrour Khenchela University, BP 1252 Road of Batna, Khenchela 40004, Algeria; anis.bertella@univ-khenchela.dz; 2 Department of Bioinformatics, National Institute of Research and Development for Biological Sciences, 296 Splaiul Independentei, Sector 6, 060031 Bucharest, Romania; georgi.gavril@yahoo.com; 3 Departmento de Química Analítica, Instituto de Investigación en Ingeniería de Aragón (I3A), Escuela de Ingeniería y Arquitectura (EINA), Universidad de Zaragoza, María de Luna 3 (Edificio Torres Quevedo), 50018 Zaragoza, Spain; magdalenka.wrona@gmail.com (M.W.); fjsl@unizar.es (J.S.); cnerin@unizar.es (C.N.); 4 Faculty of Health Sciences, San Jorge University, Villanueva de Gállego, Autovía A-23 Zaragoza-Huesca Km. 299, 50830 Zaragoza, Spain; 5 Faculty of Sciences and Technology, Department of Biology, Amine Elokkal El Hadj Moussa Egakhamouk University of Tamanghasset, Tamanghasset 11000, Algeria; a.sidaoui@univ-tam.dz; 6 Laboratory of Applied Microbiology, Department of Biology, Faculty of Life and Nature Sciences, University of Oran 1 Ahmed BenBella, Oran 31100, Algeria; benlahcen.kh@gmail.com (K.B.); kihalm@gmail.com (M.K.); 7 Physical Chemistry and Physicochemistry of Polymers, Faculty of Chemistry, Nicolaus Copernicus University in Toruń, Gagarin 7 Street, 87-100 Toruń, Poland; olewnik@umk.pl

**Keywords:** aromas, antioxidants, *Artemisia campestris*, *Artemisia herba-alba*, *Salvia jordanii*, odor activity value

## Abstract

In samples of *Artemisia campestris* (AC), *Artemisia herba-alba* (AHA) and *Salvia jordanii* (SJ) essential oils, up to 200 distinct volatile compounds were identified. Using headspace solid-phase microextraction combined with gas chromatography–olfactometry–mass spectrometry (HS-SPME-GC-O-MS), different panelists detected 52 of these compounds. This study offers the most detailed analysis of bioactive compound profiles conducted so far. The most abundant compounds identified were curcumene, making up 12.96% of AC, and camphor, constituting 21.67% of AHA and 19.15% of SJ. The compounds with the highest odor activity value (OAV) were *(E,Z)*-2,4-nonadienal (geranium, pungent), 3-nonenal (cucumber) and 2-undecenal (sweet) in AC, AHA and SJ, respectively. AHA essential oil showed significant antioxidant activity (IC_50_ = 41.73 ± 4.14 mg/g) and hydroxyl radical generation (hydroxylation percentage = 29.62 ± 3.14), as assessed by the diphenylpicrylhydrazyl (DPPH) method. In terms of oxygen radical absorbance capacity (ORAC), the strongest antioxidant activity was obtained for SJ essential oil (antioxidant activity of the essential oils, AOX = 337.49 ± 9.87).

## 1. Introduction

The use of natural antioxidants in the food industry is gaining considerable attention [1] due to their excellent safety, in contrast to the many synthetic antioxidants that are currently approved as food additives and are added during or at the end of food processing to prevent oxidation. These synthetic antioxidants are undesirable due to their toxicity and health-damaging activity. In addition, international legislation is increasingly restricting their use. Therefore, the search for natural antioxidants from plants as an alternative to synthetic antioxidants is of great interest to scientists today [2,3,4,5,6,7].

Foods are exposed to both microbiological and chemical degradation reactions during their preparation, distribution, and storage. Oxidation is one of the main factors limiting the shelf life of foods. It can occur even in foods containing less than 1% lipids [8]. Oxidation affects the organoleptic properties and causes a loss of nutritional value through the degradation of essential fatty acids and the fat-soluble vitamins A, D, E and K [9]. The study of essential oils from wild edible plants is highly desirable due to their aroma profile and functional properties (antioxidant, antimicrobial or anti-inflammatory), which can be applied in many fields such as the pharmaceutical, cosmetic and food industries [10]. In particular, essential oils have been shown to have significant antioxidant properties [11]. Therefore, there has been increasing interest in their use as natural antioxidants and their addition to foods. They have also been incorporated into active packaging and edible coatings to extend the shelf life of foods [12,13,14,15,16].

*Artemisia campestris* and *Artemisia herba-alba* are shrub species of the genus Artemisia distributed in the western Mediterranean [17,18]. *Salvia jordanii* is a woody plant, one of two species of the genus formerly named *Rosmarinus*, found only in Andalusia (Spain) and on the coasts of North Africa [19]. Essential oils from these three plants have been reported to have interesting antioxidant activity [17,18,19]. As these plants have a limited geographical distribution, they are expected to have a unique fingerprint of aromatic bioactive compounds [20]. It should be highlighted that the Mediterranean region, known for its biodiversity and the richness of its endemic plant species, provides a unique ecological context for these plants. Their adaptation to specific environmental conditions may have led to the development of unique secondary metabolites, which could offer new insights into antioxidant mechanisms and applications in various industries. Furthermore, the investigation into these species contributes to the broader understanding of Mediterranean flora’s phytochemical diversity and its conservation. As these plants have a limited geographical distribution, their study also emphasizes the importance of preserving biodiversity and the potential it holds for discovering new bioactive compounds. By focusing on these less well-known species, this research aims to highlight the untapped potential of Mediterranean flora and underscore the need for further exploration and conservation efforts in this region.

It should be emphasized that there is a need for studies on the sensory evaluation of essential oils and active packaging, especially the odor profile, as odor is one of the most important properties of essential oils [21]. The aroma profile is based on volatile bioactive compounds, which are substances synthesized by plants as phytochemicals (secondary metabolites). Therefore, aroma bioactive fingerprint analysis consists of non-target chemical analysis, screening of volatile bioactive compounds combined with simultaneous in-depth characterization of the aroma.

The application of headspace solid-phase microextraction–gas chromatography–olfactometry–mass spectrometry (HS-SPME-GC-O-MS) allows for the complete non-target analysis of volatile compounds while paying special attention to the profiling of aromatic compounds. We are dealing here with sensory and chemical detection. In the chosen technique, two different detectors (human nose and MS) simultaneously detect odor compounds in essential oil samples. For certain compounds, a well-trained human nose may be more sensitive than the MS detector, making it possible to detect odorous compounds present at very low concentrations [22,23].

Furthermore, the combination of quantitative analysis and odor detection threshold leads to the determination of the odor activity value (OAV), which can be used as an indicator of the perception of an aromatic compound [24].

To the best of our knowledge, there is no previous work on the aroma profile, OAV or antioxidant activity of *Artemisia campestris* (AC), *Artemisia herba-alba* (AHA) and *Salvia jordanii* (SJ) essential oils presented together. In previous published studies, odorous compounds were only tentatively identified by GC-MS and retention index. However, their OAVs were not determined and therefore their contribution to the aroma profile of AC, AHA and SJ was not investigated. Furthermore, a comprehensive study of the detailed aroma fingerprints is lacking.

The primary objective of this research was to comprehensively evaluate the antioxidant potential of *Artemisia campestris*, *Artemisia herba-alba* and *Salvia jordanii* essential oils. In addition, this study focuses on identifying and analyzing the volatile compounds within these oils, with a particular emphasis on odoriferous compounds. By employing headspace solid-phase microextraction–gas chromatography–olfactometry–mass spectrometry (HS-SPME-GC-O-MS), we aimed to determine the contribution of these compounds to the overall aroma profile of the oils.

## 2. Materials and Methods

### 2.1. Chemicals

Anhydrous sodium sulfate (CAS 7757-82-6), α,α–diphenylpicrylhydrazyl radical (DPPH; CAS 1898-66-4), 2,2′-azobis(2-amidinopropane) dihydrochloride (AAPH; CAS 2997-92-4), gallic acid (CAS 149-91-7), fluorescein (CAS 2321-07-5), ≥30% hydrogen peroxide (CAS 7722-84-1), sodium salicylate (CAS 54-21-7), sodium acetate (CAS 127-09-3), acetic acid (CAS 64-19-7), 85% ortho-phosphoric acid (CAS 7664-38-2), terpinen-4-ol (CAS 20126-76-5), linalool (CAS 78-70-6), 1-phenyl-2-butanone (CAS 1007-32-5), o-xylene (CAS 95-47-6), 1-decanol (CAS 112-30-1), nonanal (CAS 124-19-6), 3,5-diethyl-2-methylpyrazine (CAS 18138-05-1), cumin aldehyde (CAS 122-03-2), coumarin (CAS 91-64-5), geraniol (CAS 106-24-1), caryophyllene oxide (CAS 1139-30-6), pentadecane (629-62-9), 5-methylfurfural (CAS 620-02-0), methyl benzoate (CAS 93-58-3), benzaldehyde (CAS 100-52-7), (E,Z)-2,4-nonadienal (CAS 5910-87-2), carvacrol (CAS 499-75-2), menthol (CAS 2216-51-5), valeric acid (CAS 109-52-4), eugenol (CAS 97-53-0), methyl eugenol (CAS 93-15-2) and C7-C40 Saturated Alkanes Standard certified reference material, 1000 μg/mL each component in hexane were from Sigma Aldrich (Madrid, Spain).

Acetone (high performance liquid chromatography grade, CAS 67-64-1), ethanol absolute (GC-MS, CAS 64-27-5) and methanol (liquid chromatography-MS, CAS 67-56-1) were from Scharlab (Barcelona, Spain). Ultrapure water was obtained from a Wasserlab Ultramatic GR system (Barbatáin, Spain).

### 2.2. Samples

Samples, consisting of approximately 2 kg of *Artemisia campestris*, *Artemisia herba-alba* and *Salvia jordanii* aerial parts, were collected at the flowering stage in October and November (2015), at an altitude of 988 m in Algeria (GPS coordinates: latitude 35°35′55.707″ N, longitude 6°12′53.942″ E). The species was confirmed at the herbarium of the University of Oran 1—Ahmed Ben Bella, Algeria.

### 2.3. Sample Preparation

A representative sample consisted of 200 g of dried aerial parts from each plant. All plants were dried at 25 °C in a dark and inert atmosphere for 20 days. All samples were then ground using a cross beater mill (Retsch SK 100) from Retsch France Verder S.A.R.L. (Éragny, France). The particle size was set at 2 mm. Samples were ground to increase extraction surface area and, thus, the extraction yield. The hydrodistillation of the dried samples was performed with a Clevenger type apparatus for 3 h. As a result, the essential oil of each plant was obtained, and the extraction was performed twice. Anhydrous sodium sulfate was used to dry the essential oils obtained, which were then stored in sealed amber vials at 4 °C until further analysis. The essential oil yields were 0.64, 0.29 and 0.36% (*w*/*w*) for *Artemisia herba-alba, Artemisia campestris* and *Salvia jordanii*, respectively.

**Figure 1:**
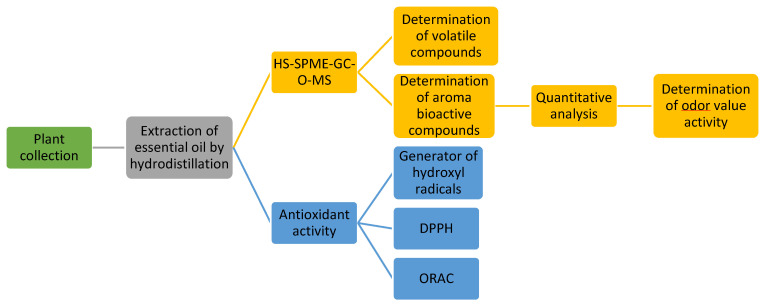
Schematic workflow chart of the performed investigation on aroma bioactive fingerprints.

A schematic workflow chart of the performed investigation on the aroma bioactive fingerprints is shown in Figure 1. It should be highlighted that bioactive aroma compounds were determined by sensory analysis. 

### 2.4. HS-SPME-GC-O-MS Conditions

For the analysis of bioactive volatiles, 25 µL of essential oil was added to 20 mL headspace SPME glass vials closed with magnetic screw caps with a polytetrafluorethylene (PTFE)/silicone septum. The vial was then placed in a heating block and equilibrated at 40 °C for 2 min. Adsorption of compounds was then performed using 100 μm polydimethylsiloxane (PDMS) fiber from Supelco (Bellefonte, PA, USA) at 40 °C for 15 min. The fiber was manually desorbed in a GC injector (250 °C) for 2 min. 

HS-SPME-GC-O-MS analysis was performed on a 7890N gas chromatograph system with a 5977D mass detector from Agilent Technologies (Santa Clara, CA, USA). Chromatographic separation was performed on a HP-5ms column (30 m × 0.25 mm × 0.25 µm) supplied by Agilent (Madrid, Spain). The following oven temperature program was used: the initial temperature was set at 40 °C (5 min), then increased to 216 °C at 7 °C/min. It was then raised again to 300 °C at 30 °C/min and held for 2 min. The gas carrier was helium at a flow rate of 2 mL/min. The injector temperature was 250 °C. The acquisition mode was SCAN in the range of 45–350 *m*/*z*. The temperatures of the MS (mass spectrometer) source and the quadrupole were set at 230 °C and 150 °C, respectively.

The OP275 (Phaser, GL Sciences, Eindhoven, The Netherlands) olfactometric detector port was used for olfactometric analysis, operating with air and helium as an auxiliary gas. In addition, deionized water was added to the vial connected to the sniffer port, to avoid the drying of the nose. A 4-port column splitter was used to connect the GC column, MS detector, and sniffing port by means of fused silica capillary tubes (0.25 mm) transfer lines provided by Agilent (Madrid, Spain). Auxiliary gas flow was 5 mL/min. The temperature of the heating line was 110 °C.

### 2.5. Optimization of HS-SPME-GC-O-MS Conditions

Three different fibers were tested, including 50/30 μm divinylbenzene/carboxen/polydimethylsiloxane (DVB/CAR/PDMS; gray hub), 100 μm polydimethylsiloxane (PDMS; red hub) and 85 μm polyacrylate (PA; white hub). Different essential oil extraction temperatures (40, 50 and 60 °C) and times (15, 20 and 30 min) were tested. Extraction efficiency was based on the number and intensities of detected peaks. Prior to the first use, all fibers were conditioned according to the manufacturer’s specifications.

### 2.6. Sensory Analysis

The aroma bioactive fingerprint analysis was conducted by a panel of six experts in aroma testing. The internal panels were researchers from the laboratory (three males, whose average age was 28 years; three females, whose average age was 34 years). They were accustomed with olfactometry and natural products characterization. The chosen panel members received thorough training, which involved becoming acquainted with a variety of scents, especially those derived from plants. The training utilized standard scent kits and various concentrations of plant extract samples. The panelists smelled each sample three times and rated the aroma of each compound sensed. The tests have been performed in the sensory room which in our case consists of a special room for GC-O-MS equipment, where the perception of odors may not be disturbed by off-odors of chemicals or gases.

In conducting this study, we meticulously followed ethical standards, as outlined in the Institute of Food Science & Technology (IFST) guidelines [25], to safeguard the well-being and rights of all participants. Before participating, each individual received a comprehensive explanation about the research’s aims, methods, possible risks, and benefits. Additionally, we took great care to ensure that none of the research activities would lead to any harm or discomfort for the participants.

### 2.7. Qualitative Analysis

The National Institute of Standards and Technology (NIST; Gaithersburg, MD, USA) library (NIST14) was used to match the spectra of detected compounds and identify them (minimum quality 80%). Additionally, for every compound identified in the samples, the retention index (RI) was determined. This calculation was performed using a standard range of n-alkanes (C7–C40) and conducted under identical chromatographic conditions.

In addition, all available pure standards were injected, to confirm the detected compounds. Then, the retention index, together with the aroma type, was used for their identification, using the Flavornet [26] and Pherobase [27] databases.

### 2.8. Semi-Quantitative Analysis

External calibration by gravimetric control was chosen for semi-quantitative analysis. All compounds were quantified against terpinen-4-ol (central peak of the chromatograms). The 20 µL of standard prepared in ethanol were placed in 20 mL glass vials and processed according to the HS-SPME-GC-O-MS method previously described. Samples were diluted 40 times with ethanol to allow the quantification of compounds present at very high concentrations.

In addition, analytical parameters, such as linearity and limits of detection and quantification (LOD and LOQ), were determined. The signal-to-noise (*s*/*n*) method was used to determine the LOD (s/*n* = 3) and LOQ (s/*n* = 10).

### 2.9. Odor Activity Values (OAVs)

The OAVs values were calculated using Equation (1):

(1) $$OAV=\frac{C_{CO}}{C_{OT}}$$

where C_CO_ is the concentration of the compound (µg/g) and C_OT_ is the odor detection threshold of the compound (µg/g), compiled from various references [24,28,29,30,31,32]. C_OT_ represents the lowest concentration at which half of the experts in aroma testing are able to smell and detect the odor [33]. 

Most of the aromatic compounds determined by HS-SPME-GC-O-MS did not provide a characteristic peak but were smelled by the panelists. Therefore, the OAV of analytes without a chromatographic peak was calculated using the LOD. In the absence of an analyte standard, a compound with a similar chemical structure was used. If there was no similar standard, the central peak of the chromatogram (linalool) was used for calculations. All standards and their LOD are shown in Table 1.

### 2.10. Antioxidant Activity

#### 2.10.1. Generator of Hydroxyl (OH·) Radicals

The antioxidant capacity of the essential oils was measured with free radical scavenging activity, using an in situ gas-phase hydroxyl radical generator, according to the technique and apparatus developed by Pezo et al. [1]. The aerosol was generated using the nebulizer connected to a Bio-Rad peristaltic pump (Hercules, CA, USA), set at a flow rate of 0.8 mL/min. The total air flow rate was set at 4.20 L/min. The OH· free radicals were generated from hydrogen peroxide (0.8%) using UV lamps (Philips Eindhoven, The Netherlands) for the photochemical reaction. The apparatus consists of eight Pasteur pipettes connected to the radical generator and to amber glass bottles.

An amount of 0.04 g of essential oil was added to the Pasteur pipette containing glass wool (0.3 g). We placed 50 g of 2 µg/g aqueous sodium salicylate solution at pH 4.5 in 100 mL amber glass bottles. Phosphoric acid (1 µg/g) was used for pH adjustment. The chromatographic analysis of 2,5-dihydroxybenzoic acid (2,5-DHB) and residual salicylic acid was performed on an HPLC system (Waters 2795, Milford, MA, USA) coupled to a fluorescence detector (Waters 474) operating at the optimal wavelengths for both compounds (λ_ex_ = 324 nm, λ_em_ = 448 nm). The separation was performed on a reversed-phase column (Waters XTerra MS C18). The mobile phase was aqueous acetate buffer (35 mmol/L, pH 5.9) and methanol, 90/10 (*v*/*v*) in isocratic mode (1.0 mL/min). The injection volume was 10 μL. If the essential oil scavenges the free radicals, the fluorescent hydroxylated 2,5-DHB is not formed and, therefore, the percentage of hydroxylation is lower than that of the blank. Results are expressed as percent hydroxylation.

#### 2.10.2. Diphenylpicrylhydrazyl (DPPH)

The antioxidant activity of the essential oils was evaluated by the DPPH method, as described by Akrami et al. [34], with some modifications. The analysis was performed by preparing different concentrations of essential oils in methanol (25.0, 12.5, 10.0, 5.0 and 2.5%). The reaction was performed by adding 100 μL of each dilution to 3.5 mL of DPPH solution (30 μg/g in methanol). The blank was also measured with 100 μL methanol. All samples were kept in the dark for 30 min. After this time, the absorbance of the samples was measured at 515 nm using a Shimadzu UV-1700 PharmaSpec spectrophotometer (Duisburg, Germany). The concentration of DPPH was checked by external calibration (4–64 μg/g). DPPH solution was prepared daily.

The antioxidant capacity of the samples was expressed as the percentage inhibition of DPPH (I%) and calculated according to Equation (2), as follows:

(2) $$I\%=\frac{A_{0}-A}{A_{0}}\times 100$$

where *A*_0_ and *A* are the absorbance values of the blank sample (DPPH with methanol) and the essential oil sample (DPPH with essential oil), respectively. The curve of the percentage inhibition values after 30 min versus the concentration of the essential oil was plotted, and linear regression was calculated to obtain the IC_50_ value (the half maximal inhibitory concentration—concentration that gives a 50% reduction in the concentration of DPPH). The obtained IC_50_ value is inversely proportional to the antioxidant activity. The results were compared with the antioxidant activity of gallic acid (positive control) preformed according to the same procedure.

#### 2.10.3. Oxygen Radical Absorbance Capacity (ORAC)

The antioxidant activity of the essential oils (AOX) was measured according to an ORAC assay, adapted from and described by Bentayeb et al. [35], which is based on the reaction of 2,2′-azobis (2-amidinopropane) dihydrochloride radical and fluorescein. The 1 h fluorescence decay was determined in the HPLC system already described in Section 2.10.1 (generator of hydroxyl radicals). For that, 100 μL of diluted essential oil in acetone or a blank sample (acetone only) was added to 800 μL of fluorescein solution. The reaction was started by adding 600 μL of AAPH reagent. Then, 20 µL of the mixture were injected every minute using a 0.5 mL/min water flow; the reaction was carried out in a thermostatic autosampler set at 40 °C. Excitation and emission wavelengths were set up at 540 and 565 nm, respectively. Fifty injections were conducted for each assay, describing the fluorescein decay. The area under the curve (AUC) was calculated according to Equation (3), as follows:

(3) $$AUC=\left(\frac{f_{1}}{f_{0}}+\frac{f_{2}}{f_{0}}\cdots +\frac{f_{i}}{f_{0}}+\cdots \right)\times \Delta t$$

where *f*_0_ is the first peak area, *f_i_* is the area of the peak *i* and Δ*t* is the time interval between consecutive peaks. The net AUC was obtained by subtracting the AUC of the blank from that of the sample.

Serial concentrations of 0, 50, 100, 200 and 250 μg/g of Trolox were prepared to obtain the calibration curve. The final ORAC values were calculated using a regression equation (Trolox concentration vs. net AUC). The results were expressed as µg of Trolox equivalents as per gram of essential oil and μmol of Trolox per g of essential oil.

### 2.11. Statistics

All experiments were performed at least in triplicate. The results were expressed as mean ± standard deviation. The statistical significance between different essential oils in different analysis was evaluated by a one-way ANOVA test followed by a Student’s *t*-Test at a significance level of *p* < 0.05.

## 3. Results and Discussion 

### 3.1. Qualitative and Semi-Quantitative Analysis

Essential oils are complex mixtures of secondary metabolites. Many plant extracts contain volatile compounds that are responsible for their aroma and flavor characteristics. These volatiles, when included in packaging, could potentially impact the sensory attributes of the packaged food. Moreover, OAV becomes a crucial factor when considering the sensory impact of packaging on food. If the plant extracts used in the packaging have a high OAV, they may impart a noticeable aroma to the food product. This could be desirable in some cases (enhancing aroma) or undesirable (overpowering the food’s natural aroma) depending on the nature of the food and the consumer’s preference. In active packaging, the balance between the antioxidant functionality and the sensory impact (flavor and aroma) of the plant extracts is key. While the primary goal is to utilize the antioxidant properties to preserve the food, the volatile compounds within these extracts and their contribution to the overall aroma and flavor profile (as indicated by their OAV) must also be considered.

Two hundred and four different volatile compounds in all three essential oils samples of AHA, AC and SJ collected in Algeria have been detected. Among them, one-hundred and fifty-three compounds were detected by MS detector and fifty-two by panelists using the sniffing port. Only five compounds, namely eugenol, isobornyl formate, *(E)*-2-nonenal, perillene and caryophyllene oxide, were detected by both detectors. It suggests that analysis using only MS without olfactometry does not provide the full profile of the odorous compounds of essential oils.

After the analysis of the data from Table 2, it can be concluded that the largest chemical group of found compounds are terpenes. They are a huge and varied class of organic compounds derived from isoprene (2-methylbuta-1,3-diene), a hydrocarbon containing five carbon atoms. It has been shown that volatile terpenes play defensive role in plants [36]. In addition to their important biological role, plant-derived terpenes are widely used in industry as natural fragrances and aromas, pharmaceuticals, cosmetic ingredients, insecticides and food additives, among others. Moreover, terpenes have been shown to possess antioxidant activity preventing oxidative damage, which makes them very attractive active agents for food industry [37]. It should be highlighted that all these areas have tremendous commercial value.

The relative percentages were calculated using data from Table 2. The highest relative percentages of curcumene (12.96%), 2-epi-*(E)*-beta-caryophyllene (12.62%), gamma-terpinene (7.66%) and alpha-pinene (7.50%) were detected in AC essential oil, while AHA essential oil is characterized by the highest relative percentages of camphor (21.67%), 3-thujanone (17.11%), gamma-muurolene (11.84%) and eucalyptol (7.67%). Finally, camphor (19.15%), limonene (14.23%), 4-carene (10.12%), (1S)-beta-pinene (6.23%) and alpha-pinene (5.87%) were identified as the major volatile compounds of SJ essential oil. All compounds have been listed in descending order, using concentrations expressed as percentage of total volatile compounds.

The qualitative analysis of AC, AHA and RC essential oils presented to date in the literature result in an extensive list of terpenes [17,19,38]. Nevertheless, the amount of detected volatile compounds was significantly lower than those listed in this study. Moreover, the literature reports a high content of beta-pinene (36.4%) and 2-undecanone (14.7%) in AC essential oil [17]. A high content of camphor (32.3%) and chrysanthenone (25.6%) have been detected in AHA essential oil [39], while beta-amyrin (17.7%) and camphor (16.9%) have been demonstrated to be the main constituents in the CO_2_ supercritical fluid extract of SJ leaves [40]. This variation could be attributed to the region of cultivation of the plant, which can influence the chemical composition of the essential oil [18].

Analytical parameters for the applied chromatographic method for terpinen-4-ol standard used for semi-quantification were as follows: LOD = 0.82 ng/g; LOQ = 2.74 ng/g; r = 0.9999; and linear range 0.0027–22.19 μg/g. A very strong linear correlation coefficient in the wide range of concentrations was obtained.

**Table 2:** Results of qualitative and quantitative analysis *.

No	Compound	Ret. Index (RI)		Concentration (μg/g)		
		Calc.	Adams (** NIST)	AC	AHA	SJ
1	1,2,5,5-tetramethyl-1,3-cyclopentadiene	839	835**		2.42 ± 0.07	
2	santolina triene	918	906		8.11 ± 0.49	
3	tricyclene	931	921		3.93 ± 0.04	7.37 ± 0.73
4	alpha-pinene	946	932	110.21 ± 0.54	20.65 ± 0.82	119.18 ± 4.09
5	camphene	966	946	4.60 ± 0.55	59.20 ± 2.07	
6	beta-thujene	986	966 **		5.23 ± 1.21	
7	beta-pinene	989	974	71.46 ± 1.39	16.96 ± 0.35	8.66 ± 0.13
8	beta-myrcene	1014	988 (994 **)	56.66 ± 2.70		28.63 ± 1.71
9	(1S)-beta-pinene	1007	989 **			126.55 ± 4.21
10	4-carene	973	993 **			205.55 ± 3.33
11	mesitylene	1005	994		26.32 ± 1.28	
12	psi-limonene	1027	1010 **			7.75 ± 0.90
13	1,2,4-trimethylbenzene	1017	1021		14.78 ± 1.62	
14	eucalyptol	1055	1035 **		138.59 ± 0.16	18.64 ± 0.76
15	limonene	1055	1044 **			288.88 ± 11.17
16	1,5-dimethyl-1,5-cyclooctadiene	1059	1047 **	23.42 ± 1.24		
17	trans-beta-ocimene	1066	1052 **	71.13 ± 2.61		
18	gamma-terpinene	1053	1054	112.49 ± 1.08		16.46 ± 1.95
19	beta-terpinene	1076	1056 **		20.26 ± 1.03	10.13 ± 0.16
20	alpha-ocimene	1077	1057 **	34.27 ± 0.98		
21	terpinolene	1110	1086 (1090 **)	2.39 ± 0.26		4.97 ± 1.37
22	isoterpinolene	1095	1090 **	1.41 ± 0.25		
23	2-isopropyl-5-methyl-2-hexenal	1089	1100 **	52.27 ± 8.91		53.83 ± 1.98
24	perillene	1120	1102	3.34 ± 0.16		<LOD
25	cis-p-menth-2-en-1-ol	1088	1106 **			22.32 ± 3.89
26	filifolone	1123	1107 **	2.40 ± 0.96		
27	p-xylene	1102	1110 **			29.71 ± 2.97
28	3,4-dimethylbenzyl alcohol	1109	1113 **			20.89 ± 2.26
29	(*E,E*)-allo-ocimene	1103	1121 **	8.86 ± 1.21		
30	allo-ocimene	1106	1128 (1113 **)			24.44 ± 1.91
31	3,4-dimethyl-2,4,6-octatriene	1125	1131 **			6.00 ± 0.17
32	cosmene	1136	1130 **	1.35 ± 0.31		
33	(*E*)-2,6-dimethyl-1,3,5,7-octatetraene	1140	1134 **	1.26 ± 0.38		
34	trans-pinocarveol	1163	1135 (1155 **)	9.72 ± 0.11		
35	camphor	1167	1141 (1161 **)	1.64 ± 0.15	391.27 ± 4.43	388.82 ± 13.09
36	(*E*)-2-nonenal	1175	1157 (1166 **)	1.73 ± 0.14		
37	3-thujanone	1148	1158 **	15.17 ± 1.99	309.04 ± 2.76	3.47 ± 0.01
38	p-mentha-1,5-dien-8-ol	1186	1166	1.81 ± 0.58		
39	albene	1179	1167 **	1.90 ± 0.24		
40	3-thujen-2-one	1191	1171	4.97 ± 0.63		
41	alpha-terpineol	1210	1186 (1190 **)	2.59 ± 0.77		45.19 ± 1.71
42	L-alpha-terpineol	1211	1192 **	4.78 ± 0.58		
43	eucarvone	1219	1199 **	2.29 ± 0.14	16.39 ± 3.49	
44	trans-dihydrocarvone	1206	1200			11.49 ± 1.55
45	2-pinen-4-one	1234	1204 (1214 **)		24.45 ± 1.04	
46	isobornyl formate	1258	1223 (1244 **)			1.90 ± 0.81
47	(*Z*)-2-(3,3-dimethylcyclohexylidene)ethanol	1229	1225 **			26.98 ± 5.28
48	cis-carveol	1254	1226 (1241 **)			2.13 ± 0.29
49	cis-3-hexenyl-alpha-methylbutyrate	1246	1229		10.56 ± 1.46	
50	2-pentylcyclopentanone	1247	1230 **		8.31 ± 0.36	1.92 ± 0.17
51	6,6-dimethylcycloocta-2,4-dienone	1240	1230 **	1.99 ± 0.21	19.13 ± 0.71	
52	bornyl formate	1227	1235	1.98 ± 0.03	23.88 ± 0.44	42.87 ± 0.99
53	(*E*)-2-hexenyl pentanoate	1262	1243 **		6.23 ± 0.56	
54	1-phenyl-but-3-en-1-ol	1229	1244 **		4.69 ± 1.66	
55	benzaldehyde, 4-(1-methylethyl)-	1271	1251 **			1.64 ± 0.71
56	2-pinen-4-on	1251	1245 **	1.81 ± 0.25	13.43 ± 0.70	1.85 ± 0.28
57	trans-2-hexenyl isovalerate	1260	1245 **		2.08 ± 0.93	
58	cis-chrysanthenol acetate	1290	1261 (1277 **)		21.70 ± 1.49	
59	trans-carveol	1255	1261 **		6.10 ± 0.68	
60	alpha,alpha,4-trimethyl-3-cyclohexene-1-methanethiol	1244	1264 **			7.16 ± 0.09
61	isobornyl acetate	1257	1268 **			1.83 ± 0.31
62	D-carvone	1279	1270 **		11.60 ± 1.78	
63	hexyl n-valerate	1258	1272 **		1.96 ± 0.10	
64	2-isopropyl-5-methyl-3-cyclohexen-1-one	1284	1275 **			3.26 ± 0.57
65	*(Z)*-3-hexenyl valerate	1253	1279 (1236 **)		2.01 ± 0.35	
66	cumin aldehyde	1270	1226 (1250 **)			1.69 ± 0.32
67	bornyl acetate	1304	1284	2.41 ± 0.83		
68	cuminol	1273	1284 **		22.97 ± 0.34	
69	trans-bornyl acetate	1298	1289 **		7.68 ± 0.65	
70	carvacrol	1311	1298		36.91 ± 3.85	
71	2-ethyl-4,5-dimethyl-phenol	1323	1305 **			3.04 ± 0.72
72	2-hydroxypiperitone	1329	1309 **		3.05 ± 0.67	
73	(*E*)-hex-3-enyl (*E*)-2-methylbut-2-enoate	1336	1319 **		4.79 ± 0.36	
74	2,4-decadienal	1327	1320 **		4.81 ± 0.32	
75	myrtenyl acetate	1318	1324	2.42 ± 0.84		
76	*(Z)*-hex-3-enyl *(E)*-2-methylbut-2-enoate	1343	1325 **	6.60 ± 0.94		
77	hexyl *(E)*-2-methylbut-2-enoate	1347	1331 **	4.02 ± 0.30		
78	p-thymol	1322	1332 **	2.45 ± 0.62		
79	(-)-dihydrocarvyl acetate	1316	1335 **			42.91 ± 3.67
80	1,5,5-trimethyl-6-methylene-cyclohexene	1355	1338 **	4.06 ± 0.27		5.71 ± 1.53
81	alpha-cubebene	1357	1345		45.46 ± 5.17	
82	3-allylguaiacol	1365	1362 **	2.53 ± 0.03		
83	cis-chrysanthenyl propionate	1368	1355 **	8.25 ± 1.84		
84	eugenol	1389	1373 **	7.26 ± 0.06	10.28 ± 0.02	
85	alpha-ylangene	1398	1373 (1406 **)			23.27 ± 2.08
86	alpha-copaene	1403	1374 (1423 **)	44.61 ± 5.89		31.68 ± 1.74
87	alloaromadendrene	1376	1386 **		12.37 ± 1.66	
88	beta-cubebene	1362	1387 (1371 **)	5.21 ± 0.63		5.81 ± 0.44
89	(-)-beta-bourbonene	1407	1388			8.12 ± 1.09
90	3-allyl-6-methoxyphenol	1382	1392 **			5.49 ± 1.50
91	cyperene	1419	1398 (1399 **)	6.25 ± 0.97		
92	longifolene	1410	1407		34.41 ± 0.76	
93	caryophyllene	1428	1408	43.67 ± 6.64		57.51 ± 7.74
94	beta-ylangene	1399	1419			20.19 ± 0.98
95	alpha-farnesene	1439	1422 **	3.10 ± 0.06	21.98 ± 1.21	
96	1,4-dimethylnapthalene	1404	1424 **	9.36 ± 0.87	37.09 ± 3.78	7.63 ± 1.66
97	2,6-dimethylnaphthalene	1408	1426 **	43.18 ± 9.20		
98	beta-copaene	1432	1430	15.22 ± 2.14		4.08 ± 0.75
99	beta-gurjunene	1445	1431			2.61 ± 0.11
100	calarene	1431	1432 **			29.35 ± 2.62
101	trans-bergamotene	1443	1432			15.55 ± 1.93
102	aromandendrene	1437	1436 **	14.49 ± 2.77		7.93 ± 0.41
103	cis-beta-farnesene	1438	1440			7.53 ± 1.31
104	alpha-neoclovene	1438	1452	17.34 ± 3.38		
105	humulene	1447	1452	12.7 ± 1.09	9.86 ± 0.56	
106	beta-farnesene	1436	1454		11.12 ± 1.50	
107	alloaromadendrene	1450	1455 **		9.15 ± 0.44	2.88 ± 0.19
108	2-epi-*(E)*-beta-caryophyllene	1449	1463 **	185.37 ± 0.14		
109	cis-muurola-4(15),5-diene	1446	1465			1.93 ± 0.40
110	gamma-muurolene	1430	1478 (1456 **)		213.86 ± 9.78	
111	curcumene	1517	1479 (1510) **	190.40 ± 11.99		
112	alpha-amorphene	1503	1483			50.38 ± 4.84
113	germacrene D	1510	1484 (1490 **)		48.05 ± 0.33	2.42 ± 0.79
114	isopropyl cinnamate	1505	1485 **		6.61 ± 1.03	
115	beta-selinene	1520	1489	25.36 ± 2.81		8.00 ± 0.28
116	zingiberene	1519	1493	23.49 ± 1.19		
117	gamma-amorphene	1511	1495			8.37 ± 0.10
118	alpha-muurolene	1518	1500		13.46 ± 0.55	
119	bicyclogermacrene	1523	1500	23.31 ± 2.64		
120	beta-bisabolene	1517	1505			13.01 ± 0.48
121	2-isopropyl-5-methyl-9-methylenebicyclo[4.4.0]dec-1-ene	1528	1510 **			66.07 ± 3.21
122	gamma-cadinene	1523	1513	13.66 ± 0.60	5.02 ± 0.06	15.76 ± 0.33
123	viridiflorene	1503	1520 **		4.74 ± 0.53	
124	4-ethylbenzoic acid, but-3-yn-2-yl ester	1525	1521 **		7.02 ± 0.41	
125	sigma-cadinene	1529	1524 **	3.11 ± 0.90	15.77 ± 0.54	
126	zonarene	1526	1528	15.23 ± 2.31		
127	nerolidol isomer 1	1544	1531	3.17 ± 0.21		
128	cubenene	1535	1532 **	6.04 ± 0.21		5.20± 0.27
129	italicene ether	1537	1536	2.53 ± 0.26		
130	alpha-cadinene	1533	1537			5.16 ± 0.10
131	alpha-calacorene	1539	1544	2.56 ± 0.08	3.24 ± 0.21	
132	nerolidol isomer 2	1546	1561	6.87 ± 1.42		
133	cis-3-hexenyl benzoate	1551	1565	4.82 ± 1.32		
134	caryophyllene oxide	1545	1582 (1549 **)		4.97 ± 0.08	
135	neryl (S)-2-methylbutanoate	1608	1582	28.90 ± 1.72		
136	caryophyllene oxide isomer 2	1614	1596 **	<LOD		4.10 ± 0.12
137	isoaromadendrene epoxide	1617	1594 **	9.94 ± 0.78	16.43 ± 0.07	
138	alpha-humulene epoxide II	1638	1608 (1620 **)			1.20 ± 0.02
139	globulol	1624	1610 **	7.14 ± 1.00		
140	isospathulenol	1601	1621 **		5.85 ± 1.13	
141	trans-carvyl *(E)*-2-methyl-2-butenoate	1622	1631		11.18 ± 0.21	
142	tau-cadinol	1667	1638 (1660 **)			2.28 ± 0.03
143	isoaromadendrene epoxide	1644	1639	3.38 ± 0.69		
144	alpha-eudesmol	1656	1643 **	2.48 ± 0.33		
145	7-methyl-1,8-naphthyridin-2-amine	1626	1644 **		3.60 ± 0.20	
146	di-epi-1,10-cubenol	1655	1645			1.19 ± 0.04
147	cubenol	1660	1651 **		2.47 ± 0.79	
148	alpha-cadinol	1665	1652			1.58 ± 0.29
149	aromadendrene oxide-(2)	1647	1678 **	4.19 ± 0.52	2.25 ± 0.94	
150	eudesm-7(11)-en-4-ol	1680	1681			2.60 ± 0.95
151	alpha-bisabolol	1706	1686			11.54 ± 0.30
152	8-cedren-13-ol	1671	1688		3.83 ± 0.20	
153	eudesm-7(11)-en-4-ol	1684	1700			3.82 ± 0.08

* Table 2 presents a comprehensive list of volatile (non-odorous) compounds identified in all samples. These compounds are organized in ascending order based on their retention index (RI) values, as reported in the literature. In instances where two RI values are available, the compounds are sorted according to the first RI value listed. The literature RI has been taken from Adams [41] or NIST WebBook [42]. If the compounds were not included in Adams’ list, or if the differences between the experimental RI and the values from the table exceeded 20, the NIST WebBook was consulted, and the RI was reported with a double asterisk (**).

### 3.2. Aroma Bioactive Fingerprint Evaluation

Up to fifty-two compounds were found to be aroma-bioactive, which were mainly dominated by different aldehydes and ethers. The functional group of the molecule is one of the key parameters of the odor quality. Thus, the odor threshold of a compound depends on a change in the functional group of compounds with a similar structure [43].

The contribution of those fifty-two odorous compounds to the overall aroma profile of AC, AHA and SJ essential oils was estimated by calculating their OAV according to their semi-quantified concentrations, or by the LODs and thresholds given in the literature. At the same time, the dominant active aromas were selected as compounds with OAV > 1. The compounds with the highest OAV in case of AC were *(E,Z)*-2,4-nonadienal (geranium, pungent), *(E)*-2-nonenal (cucumber, fat, green), 4-mercapto-4-methyl-2-pentanol (flower, lemon), ethyl isobutyrate (sweet) and eugenol (clove, honey). For AHA, the compounds with the highest OAV were 3-nonenal (cucumber), eugenol (clove, honey) and isogeraniol (rose). Finally, the compounds characterized by the highest OAV in case of SJ were 2-undecenal (sweet), eugenol (clove, honey) and caryophyllene oxide (herb, sweet, spice).

Literature about the aroma profiles of AC, AHA and SJ essential oils has not been found, and therefore no data comparison has been performed.

It should be highlighted that the detailed definitions of the aroma profiles of AC, AHA and SJ essential oils could be successfully used as a fingerprint for the recognition and authentication of analyzed plants.

The list of identified odorous compounds in all samples is shown in Table 3.

**Figure 2:**
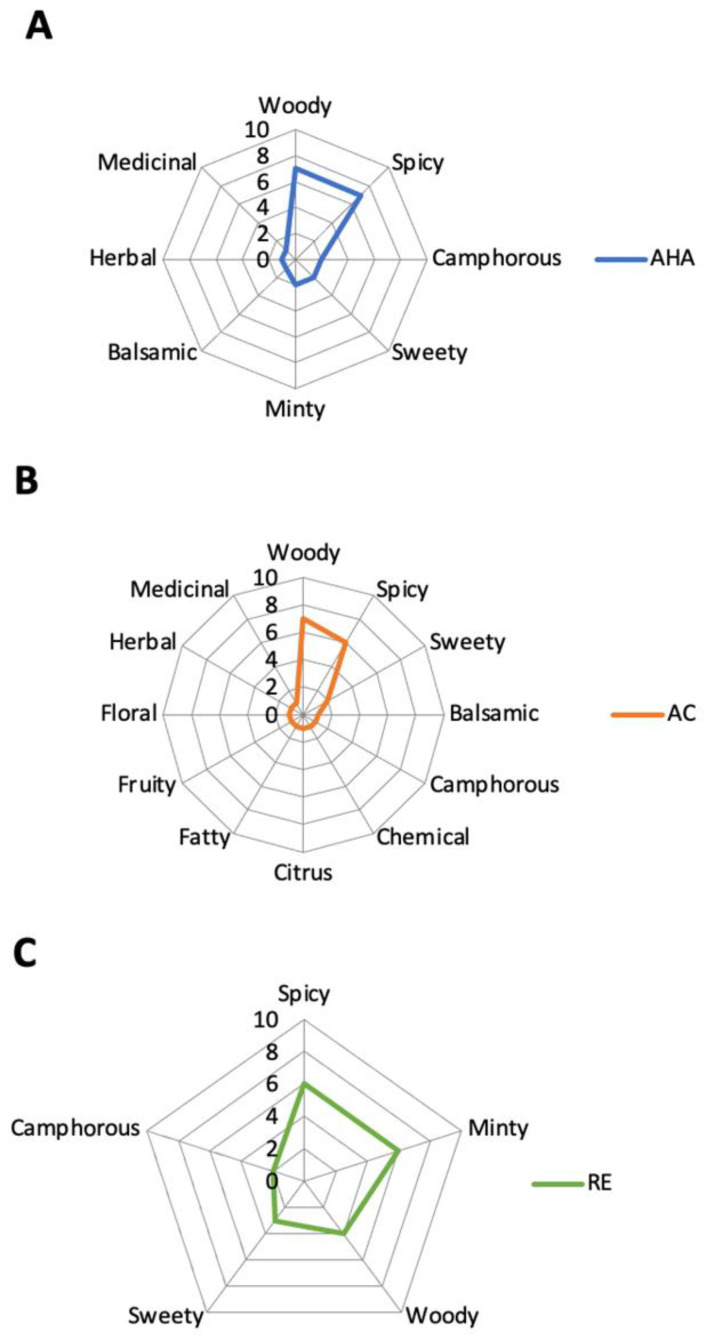
Spider graphs of the distribution of odorous classes in analyzed samples of the following essential oils: (**A**) *Artemisia herba-alba*; (**B**) *Artemisia campestris*; and (**C**) *Salvia jordanii*.

The detected odorous compounds were classified according to the 25 different classes of aromas suggested by Flavornet database [www.flavornet.org (accessed on 12 December 2023)]. The aroma spider graphs of analyzed essential oils were drawn according to the qualitative analysis of the olfactometry results, precisely by regrouping all the compounds having the same aroma descriptor into groups (Figure 2).

According to the HS-SPME-GC-O-MS results, the descriptors of aroma in AHA essential oil were classified into woody, spicy, camphorous, sweety, minty, balsamic, herbal, medicinal, chemical, citrus, fatty, fruity, and floral odor classes. The descriptors of aroma in AHA essential oil were classified into woody, spicy, camphorous, sweety, minty, balsamic, herbal and medicinal odor classes. The biggest aroma groups in both the AC and AHA samples were woody and spicy. On the other hand, the descriptors of aroma in the SJ essential oil were classified into woody, spicy, minty, camphorous and sweety odor classes. The biggest aroma groups in the case of the SJ sample were woody, spicy and minty. Most of the contributions to the woody aroma were terpenes, sesquiterpenes and alcohols, while other terpenes, terpenoids and esters contributed to the minty odor of the samples. Finally, epoxide and few aromatic compounds contributed to the spicy odor of the analyzed essential oils. Undoubtedly, the spider graph of AC essential oil is much more complex, with a significantly different odors profile than the SJ and AHA samples.

### 3.3. Antioxidant Activity

The results of antioxidant activity obtained by three different methods are shown in Table 4.

Depending on the considered method, different trends in antioxidant capacities can be observed. The differences in the essential oil activity from one method to another may be explained by different mechanisms, such as preventing initiation, the decomposition of peroxide, the prevention of the continuous abstraction of hydrogen, free radical scavenging, the ability to reduction, and the bond of transition ion catalysts. Therefore, it is essential to use several analytical methods and different substrates to evaluate the effectiveness of antioxidants [44].

#### 3.3.1. Generator of Hydroxyl Radicals

The results of the percentage of hydroxylation, equal to 29.62%, for the essential oil of AHA showed significant (*p* < 0.05) antioxidant activity, whereas the AC oil had a lower activity, with a hydroxylation level of 50.99%. The least efficient was SJ, which showed only slight activity, with the percentage of hydroxylation reaching 81.58%.

Comparing to the results reported by Pezo et al. [45], the antioxidant activity of AHA essential oil was lower than that of cinnamon, oregano and clove essential oils. On the other hand, its activity was much higher than that of rosemary, ginger, verbena and lemongrass essential oils, or even propolis, which had hydroxylation percentages of 37.03, 60.37, 61.11 and 40.47%, respectively. AC oil was found to be less active than most essential oils except ginger and lemongrass.

Finally, the antioxidant activity of the SJ essential oil was too weak, compared to the results previously found by Pezo et al. [45].

Opposite results were obtained for the samples of AHA and SJ, despite the presence of the same main ingredient in both samples. The high hydroxyl radical-scavenging properties of the AHA samples can be probably attributed to some other compounds present in the sample or the additive and/or synergistic antioxidant effect of different compounds. But it is also a possible antagonistic effect of the compounds present in the SJ sample. Nevertheless, such conclusions should be confirmed by further investigation into different mixtures of pure compounds detected in both samples.

#### 3.3.2. Diphenylpicrylhydrazyl (DPPH)

The results of the DPPH assay show that the essential oil of AHA demonstrates significant (*p* < 0.05) antioxidant activity, expressed as an IC_50_ of 41.73 ± 4.14 mg/g. Then, a slightly lower level of activity was observed for the AC essential oil, with an IC_50_ of 53.44 mg/g. In contrast, the essential oil of SJ showed a weak antioxidant capacity with a high IC_50_ of 108.31 mg/g. These results have been compared with the antioxidant capacity of gallic acid, which was the strongest antioxidant, with an IC_50_ of 46.01 ± 4.12 μg/g.

The value of the IC_50_ found for the AHA essential oil is higher than that found in a previous work undertaken by Rafiq et al. [46], who reported an IC_50_ of 2.33% (23.3 mg/g) in the same plant. Simultaneously, the essential oil of AC represents a lower IC_50_ than that recorded by Akrout et al. [47] which was 94.5 mg/mL, the same as for the essential oil of SJ, whose IC_50_ value was much higher than that found by Bendif et al. [19], which was 4.04 mg/mL.

The ability of essential oils to trap the DPPH radical may be due to the high percentage of oxygenates (mono and sesquiterpenes), such as camphor, 1,8-cineole, linalool and others, which are known for their antioxidant activity [48].

Such differences in results can be undoubtedly attributed to the different conditions of analysis presented in the literature. In addition to the different chemical composition [48], the comparison of the results remains unreliable because of the different parameters adopted during the analysis and the modified protocols that use different amounts and concentrations of DPPH, solvents (methanol, ethanol), amounts of essential oil and reaction times (15, 30 and 60 min) [44]. It should be highlighted that the DPPH method has not been standardized, and therefore it is difficult to find literature with the same conditions of experiments for a confident comparison.

#### 3.3.3. Oxygen Radical Absorbance Capacity (ORAC)

The ORAC test allowed us to estimate the antioxidant potential of essential oils, regarding their ability to trap free radicals. The method is based on the transfer reaction of a hydrogen atom [19]. The results obtained are shown in Table 4. It can be clearly observed that the essential oil of SJ is effective as an antioxidant chain breaking agent and has a significant (*p* < 0.05) antioxidant activity among the three essential oils studied, with an ORAC value of 337.49 µmol Trolox/g of essential oil, equivalent to 0.084 g Trolox/g of essential oil. This value is a little bit lower than that reported by Bendif et al. [19] for the same kind of species (leaves and flowers) collected also in Algeria in March 2015, which was 0.14 to 0.17 g of Trolox/g of essential oil. It should be highlighted that ORAC was not determined for the same part of plant (steam) and difference in results may be attributed to this.

Moreover, the results of ORAC are similar for AHA and SJ samples, and this is probably due to the presence of camphor, a volatile compound with the highest quantified concentration in both samples. Also, both samples included alpha-pinene in their composition, listed as one of the major volatile components.

Nevertheless, the obtained value is similar to the ORAC AOX values of several essential oils extracted from dill seeds, rosemary and basil, which are available on the market and were produced on the industrial scale [35]. Furthermore, the results obtained for SJ, AHA and AC remain higher than the values recorded for several *Juniperus* species, which did not exceed 0.027 g Trolox/g of essential oil [49]. The literature very often compares the analysis of these essential oils to those of the *Juniperus* species, in the context of food safety [50].

When comparing samples of two *Artemisia* species, the better results were obtained for AHA, due to its chemical composition (i.e., the presence of camphor and thujone) [51]. The results of ORAC obtained in this study may be justified by the presence, in significant quantities, of eucalyptol and α-pinene in SJ. Different AHA essential oils are scavengers of peroxyl radical derived from APPH, often found in lipid oxidation reactions [19].

## 4. Conclusions

Aroma bioactive fingerprint analysis of *Salvia jordanii*, *Artemisia herba-alba* and *Artemisia campestris* essential oils has been performed by HS-SPME-GC-O-MS applying two different detectors: MS and human nose. Also, their profiles, with an extensive number of volatile compounds, have been determined. The predominant perceptions of aromatic compounds were comprehensively investigated using a combination of quantitative analysis with an odor detection threshold, resulting in the determination of the odor activity values.

It has been shown that performance of analysis only by MS without olfactometry does not give the full profiles of odorous compounds of essential oils, as the human nose is able to detect compounds present below the limit of detection of the GC-MS method.

Different trends in antioxidant capacities evaluated by methods with different reaction mechanisms have been observed and related to the composition of specific essential oils. Despite the presence of the same main ingredient (camphor) in samples of AHA and SJ, the opposite results for the hydroxyl radical scavenging ability have been obtained.

It should be highlighted that odor and antioxidant capacity are the most crucial and interesting properties of essential oils. The study of these is extremely important from the point of view of the food industry, where essential oils are commonly applied as food additives or active agents in antioxidant packaging. Additionally, the detailed definitions of the aroma profiles of AC, AHA and SJ essential oils could be successfully used as a fingerprint for the recognition and authentication of the analyzed plants. Beyond their aromatic allure and antioxidant prowess, essential oils hold paramount importance for their antibacterial, antifungal, and antiviral properties, making them invaluable in a myriad of applications.

Future endeavors will focus on the application of these essential oils in active packaging, employing real food samples to assess their efficacy as natural preservatives. This will involve exploring not only their sensory impact but also their ability to inhibit microbial growth, thereby extending the shelf life of food products naturally. Additionally, further research into the cultivation techniques of these plant species could enhance their commercial viability, ensuring a sustainable supply of high-quality essential oils for various industrial applications.

## Figures and Tables

**Table 1:** List of standards and their LOD used for OAVs calculation.

No	Standard	LOD (µg/g)	Surrogate for Compounds *
1	linalool	0.047	1, 2, 3, 6, 7, 9, 10, 11, 24, 26, 27, 38, 39, 41, 46, 47, 48
2	1-phenyl-2-butanone	0.046	4, 16, 18, 37, 40, 51
3	o-xylene	0.348	5
4	decanol	0.025	12, 15, 50
5	nonanal	0.363	13, 19, 35
6	diethyl-methyl pyrazine	0.086	17
7	cumin aldehyde	0.049	28, 32
8	coumarin	0.485	33
9	geraniol	0.126	31, 43
10	caryophyllene oxide	0.019	42, 44
11	pentadecene	0.023	52
12	5-methylfurfural	0.978	8
13	methyl benzoate	0.032	14
14	benzaldehyde	0.174	21, 49
15	*(E,Z)*-2,4-nonadienal	0.098	22
16	carvacrol	0.032	25, 30, 34
17	menthol	0.115	20, 23
18	valeric acid	0.096	29
19	eugenol	0.901	36
20	methyl eugenol	0.193	45

* Numbers indicate the compounds listed in the Section 3.2 Aroma Bioactive Fingerprint Evaluation of which the OAV was calculated with the present standard.

**Table 3:** Results of odors detected by panelists using HS-SPME-GC-O-MS.

No	Compound	Ret. Index (RI) Calc.	Aroma	OAV		
				AHA	AC	SJ
1	epoxylinalool	751	sweet, woody	7.83		
2	ethyl isobutyrate	782	sweet		470	
3	methylbutanone	844	camphor			0.025
4	isopropyl butanoate	848	pungent, fruit	2.56		
5	*o*-xylene	869	geranium	7.00		
6	octadienone	966	fruit, fat, mushroom	0.16		
7	octanone	976	herb, butter, resin			9.40
8	5-methylfurfural	991	almond, caramel, burnt sugar		0.88	
9	2,4-heptadienal	1019	fried			0.50
10	*(E)*-beta-ocimene	1030	sweet, herb			1.38
11	beta-phellandrene	1052	mint, turpentine		1.18	1.18
12	3,3,6-trimethyl-1,5-heptadien-4-ol	1086	herb	NA **		
13	3-nonenal	1102	cucumber	4500		
14	methyl benzoate	1115	prune, lettuce, herb, sweet		61.54	
15	perillene *	1120	wood		NA	NA
16	4-mercapto-4-methyl-2-pentanol	1120	flower, lemon		1250	
17	*(E)*-rose oxide	1133	flower		92.00	
18	methylcyclopentapyrazine	1133	roast	NA		
19	*(E)*-2-nonenal	1166	cucumber, fat, green		4500	
20	menthol	1173	peppermint			0.12
21	ethylbenzaldehyde	1173	sweet		13.08	
22	*(E,Z)*-2,4-nonadienal	1202	geranium, pungent		5000	
23	epoxy-p-menthene	1206	mint, dill			NA
24	linalyl formate	1216	citrus, coriander	NA		
25	*(E)*-carveol	1218	caraway, solvent			0.13
26	isobornyl formate	1223	green, earth, camphor			NA
27	ethyl octenoate	1224	must, oil, fruit, pungent		NA	NA
28	cumin aldehyde	1226	acid, sharp	0.82		
29	isobutyric acid	1238	rancid, butter, cheese			0.012
30	DL-carvone	1267	mint, basil, fennel		4.78	
31	geranial	1280	lemon, mint			4.06
32	cuminic alcohol	1287	wood, herb	NA		
33	methyl quinoxaline	1297	roast, nut, fruit	0.00048		
34	dihydrocarvyl acetate	1357	mint, camphor, medicine		NA	
35	2-undecenal	1370	sweet			461
36	beta-elemene	1388	herb, wax, fresh	NA		
37	eugenol *	1389	clove, honey	1713	1210	150
38	ethyl decanoate	1399	grape	0.09		
39	beta-farnesene	1422	wood, citrus, sweet	0.54		
40	ethyl salicylate	1438	wintergreen, mint	NA		
41	linalyl butyrate	1447	pear, sweet	NA		
42	isogeraniol	1462	rose	107.50		
43	butyl octanoate	1463	fruit			NA
44	methyl eugenol	1477	clove, spice			0.23
45	citronellyl isobutyrate	1488	fruit, rose			NA
46	alpha-farnesene	1505	wood, sweet			0.54
47	methyl laurate	1510	fat, coconut			NA
48	isopropyl benzoate	1555	sweet, fruit			NA
49	caryophyllene oxide *	1614	herb, sweet, spice	0.046		10
50	tridecanol	1625	must		NA	
51	oxo-beta-ionone	1644	wood	NA		
52	7-heptadecene	1667	alkane	NA		

* Compounds presented in Table 2, also detected by MS. ** NA—data of threshold are not available, therefore OAV has not been calculated.

**Table 4:** Results of antioxidant activity for different samples and different methods.

Method	Generator of OH· Radicals	DPPH	ORAC	
Sample	Percentage of Hydroxylation	IC_50_ (mg/g)	AOX	
			(μmol Trolox/g of Essential Oil)	(g Trolox/g of Essential Oil) *
*Artemisia herba-alba*	29.62 ± 3.14	41.73 ± 4.14	309.08 ± 7.19	0.077 ± 0.002
*Artemisia campestris*	50.99 ± 3.31	53.44 ± 6.37	158.10 ± 4.69	0.039 ± 0.001
*Salvia jordanii*	81.58 ± 5.09	108.31 ± 8.01	337.49 ± 9.87	0.084 ± 0.002

* The results of ORAC are presented in two different units, for a better comparison with data available in the literature.

## Data Availability

The original contributions presented in the study are included in the article, further inquiries can be directed to the corresponding author.
